# *Tuned In* Emotion Regulation Program Using Music Listening: Effectiveness for Adolescents in Educational Settings

**DOI:** 10.3389/fpsyg.2016.00859

**Published:** 2016-06-07

**Authors:** Genevieve A. Dingle, Joseph Hodges, Ashleigh Kunde

**Affiliations:** School of Psychology, The University of Queensland, Brisbane, QLDAustralia

**Keywords:** emotion regulation, emotion awareness, music, engagement, adolescents

## Abstract

This paper presents an effectiveness study of *Tuned In*, a novel emotion regulation intervention that uses participant selected music to evoke emotions in session and teaches participants emotional awareness and regulation skills. The group program content is informed by a two dimensional model of emotion (arousal, valence), along with music psychology theories about how music evokes emotional responses. The program has been evaluated in two samples of adolescents: 41 “at risk” adolescents (76% males; *M*_age_ = 14.8 years) attending an educational re-engagement program and 216 students (100% females; *M*_age_ = 13.6 years) attending a mainstream secondary school. Results showed significant pre- to post-program improvements in measures of emotion awareness, identification, and regulation (*p* < 0.01 to *p* = 0.06 in the smaller “at risk” sample and all *p* < 0.001 in the mainstream school sample). Participant ratings of engagement and likelihood of using the strategies learned in the program were high. *Tuned In* shows promise as a brief emotion regulation intervention for adolescents, and these findings extend an earlier study with young adults. *Tuned In* is a-theoretical in regard to psychotherapeutic approach and could be integrated with other program components as required.

## Introduction

Effective emotion regulation relies on an individual’s awareness of their own and others’ emotions, the development of a lexicon to label a range of emotional states, and strategies to modulate emotions to suit the context. These abilities develop throughout childhood and adolescence, although the rate of acquisition varies across emotions and stimuli. There is some evidence that the development of emotional skills slows down during the onset of puberty – at a time when young people typically experience intense emotions and when their regulatory skills are not yet fully developed. The *Tuned In* program was designed to train young people in emotion awareness, labeling, and regulation, using music listening as an engaging and meaningful way of evoking emotions during sessions. An early trial of the program found it to enhance emotional clarity and regulation among young adults (Dingle and Fay, under review), and the current study extends on this work with an investigation of the effectiveness of *Tuned In* for enhancing emotion awareness and regulation of healthy and at-risk adolescents in educational contexts.

Most pre-school children (aged 3 to 5 years) are able to correctly identify primary emotions in facial expressions, body postures and a combination of cues (Nelson and Russell, 2011). Studies of older children and adolescents suggest that the development of facial emotion recognition may slow down in early puberty. For example, in one study participants were asked to decide if happy, sad, angry or neutral faces matched with one of four emotion words. Compared to 10 year old participants, reaction times were significantly slower for making a correct decision at 11 and 12 years of age (the approximate ages of puberty onset), then stabilized by 15 years of age (McGivern et al., 2002). Other studies have found that sensitivity to anger expressions remained relatively stable between upper childhood and puberty and then increased from middle to late puberty (Thomas et al., 2007; Lawrence et al., 2015).

Recognition of one’s own emotions doesn’t necessarily develop at the same rate as recognition of emotions in others. The development of a lexicon for affect labeling has long been considered a precursor for emotion regulation – particularly the regulation of negative emotions (Lieberman et al., 2007). The recognition of emotion words doubles in size every two years between 4 and 11 years old, but plateaus between 12 and 16 years old (Baron-Cohen et al., 2010). Adolescence and the onset of puberty is a time of great physical, social and emotional change, and the findings summarized here suggest that adolescents may find the processing and regulation of their own emotions particularly challenging.

Emotion regulation can be conceptualized as the ability to modify emotion in flexible and adaptive ways in response to social context (Campos et al., 2011). Those with better emotion regulation skills are more socially competent, have better quality friendships and show more prosocial behaviors than those with poor emotion regulation capacity (Eisenberg et al., 2007; Wranik et al., 2007). Adolescents commonly experience intense emotional states during puberty and the transition from childhood to adulthood (Casey and Caudle, 2013). At the same time, their full regulation capacity is still developing (Hannesdottir and Ollendick, 2007; Beauchaine, 2015). Emotion dysregulation is considered an important trans-diagnostic risk factor for mental disorders in adolescents (McLaughlin et al., 2011).

There are currently no interventions designed to specifically address emotion dysregulation in adolescents. Cognitive behavior therapy (CBT) is an evidence based treatment for a range of emotional disorders in children and adolescents (Weisz and Gray, 2008). However, strong emotions are rarely evoked during cognitive restructuring, and CBT has been criticized for lacking a focus on emotion dysregulation (Hannesdottir and Ollendick, 2007). Several other psychotherapy programs include emotion regulation components, such as: dialectical behavior therapy (DBT; Linehan, 1993), acceptance and commitment therapy (ACT; Hayes et al., 1999), emotion focused therapy (EFT; Greenberg and Bolger, 2001), and mindfulness based cognitive therapy (MBCT; Segal et al., 2002). These treatments seek to increase clients’ awareness and attention to their internal emotional states, and to build their tolerance of these states. The research evidence in support of these therapies with adolescents is still developing (Ritschel et al., 2015). Some of these treatments (e.g., DBT) are intensive and long term; designed to treat diagnosed personality disorders. It is unlikely that all adolescents with emotion regulation difficulties require this level of treatment or indeed can access a specialist DBT program. There is clearly room for a brief and engaging emotion regulation program for enhancing emotional skills in adolescents.

In the *Tuned In* program, music listening is used as the method for evoking and experiencing emotions in session for the purpose of emotion psycho-education and skills building. Personalized music listening has been consistently listed by 18–25 year old Australians as their number one emotion management strategy in the annual national survey of Australians Stress and Wellbeing (Australian Psychological Society, 2015). Adolescents and young adults are the biggest consumers of music, with one study reporting that young people listened with intention to an average of 18 h of music per week (Papinczak et al., 2015). Furthermore, music psychology research attests to the influence music listening has over emotional states captured in subjective ratings (Hunter et al., 2011), physiological responses like skin conductance and heart rate (Krumhansl, 2002; Sharman and Dingle, 2015), and imaging studies showing that the brain’s emotion centers are activated while listening to either pleasant and unpleasant music (Salimpoor et al., 2011; Hodges and Wilkins, 2015). Experience sampling and mobile application research shows that music-based emotion regulation differs from non-music emotion regulation in several key ways, and is a strategy that allows listeners to reach specific emotional goals (Randall and Rickard, 2014; Hides et al., 2015). Due to individual variations in musical preference, research indicates that personally selected music is more effective at evoking emotional responses than music selected by experimenters (Chafin et al., 2004; Weth et al., 2015). Therefore, participants’ own music selections are used in the *Tuned In* program.

The *Tuned In* program was designed to enhance participants’ emotional awareness, identification of emotions, and emotion regulation skills. Participants are introduced to a two dimensional model of emotion consistent with Hevner’s (1936) taxonomy of emotional adjectives in music, and most closely aligned with Russell’s (1980) circumplex model of emotion, in which the full gamut of emotions can be located in relation to two dimensions: valence (from pleasant to unpleasant) and arousal (from high to low energy). Studies using music stimuli have reliably mapped emotional responses onto this two dimensional model (Schubert, 1999; Eerola and Vuoskoski, 2013). The model provides a visual psycho-educational tool assisting participants to become proficient in identifying their current and desired state. Each *Tuned In* session is based on a different emotion, taking participants to a different co-ordinate on Russell’s model. **Table 1** shows the content of the 8 session version of the program.

**Table 1:** The Eight Session Version of *Tuned In* Used with the At-Risk Adolescent Sample.

Session	Content	Activities	Homework
(1) Welcome to *Tuned In*	Building group alliance, establishing group guidelines, brief program overview, Introduction to Russell’s Circumplex model of emotion.	Completion of pre-program surveys Place the emotion on the model.	Find a low energy, pleasant song to share for next week.
(2) Music to calm you down.	Positive valence, low arousal music. Review of Russell’s Circumplex model of emotion. Discussion of music characteristics (tempo, voice, instruments) and when to use calming music.	Imagery task Body scan Lyric analysis	Find a high energy, pleasant song to share next week.
(3) Music to power you up.	Positive valence, high arousal music. Discussion of musical characteristics and when energizing music may be helpful.	Imagery task Body scan Lyric analysis	Find a medium-to-high energy, pleasant song to share next week.
(4) Music to make you happier.	Positive valence, medium to high arousal. Discussion on extending/intensifying happy emotions through music.	Imagery task Body scan Lyric analysis	Find a low energy, unpleasant song to share next week.
(5) Music to be sad to.	Negative valence, Low arousal. Listening to music to explore feelings of sadness. Discussion on knowing when enough is enough and strategies to use when feeling sad.	Imagery task Body scan Lyric analysis	Find a high energy, unpleasant song to share next week.
(6) Music to shout to!	Negative valence, high arousal. Discussion on current strategies for coping with anger, shame, jealously and positives/negatives of these.	Imagery task Body scan Lyric analysis	Find a low to high energy, pleasant song to share next week.
(7) Music that inspires you.	Positive Valence, low to high arousal, discussion on what is inspiring and future directions.	Imagery task Body scan Lyric analysis	Continue to compile your emotion playlists!
(8) Keep on Groovin’.	Concluding comments and group discussion. Review of Russell’s Circumplex model of emotion and strategies to change mood states through music listening.	Post program surveys	Review booklets as needed.

Prior to each session, participants are asked to select a song that makes them feel the focal emotion for the next session and bring it along on their portable music device. An emotional “Tune In” procedure is conducted at the start of each session, in which participants are asked to indicate their current emotional state on the two dimensional model, and their desired emotional state (either the same or different), and what type of music listening or other emotion regulation strategy would help them to reach their desired emotional state. For example, if a participant felt flat and unmotivated at the start of the session, they would locate this emotional state as low on the arousal dimension and on the negative side of the valence dimension. They might want to feel more energized, so they would indicate a spot higher in arousal and on the positive side for valence. The participant would be asked to nominate some music that would help them to achieve this energized state, and some other strategies they could use (such as exercise) if music listening was not suitable.

Several participants’ songs are played in each session, and participants engage in three activities designed to focus their attention on the music in order to enhance their emotional responses. The first is an imagery task in which participants are instructed to draw any images that come to mind while they listen to the music. According to Holmes et al. (2008) and Holmes and Mathews (2010), imagery plays a role in many mental disorders, with cognitions in the form of mental images reported to have a greater impact on emotions than verbal representations alone. In the second task, participants are provided with an outline of a human body and instructed to illustrate where they experienced any physiological sensations in response to the music. This task is based on research suggesting that paying mindful attention to the physical sensations of emotions, without trying to change or alter them, can assist individual’s to build awareness and tolerance of their emotional experiences (Chambers et al., 2010). The third task is a lyric analysis with participants asked to listen to a piece of music while reading the lyrics in their manuals, circling or underlining any lyrics that are particularly emotionally moving for them. Participants are then invited to discuss what the lyrics meant to them, along with sharing any emotional responses they evoked. According to McFerran (2010, p. 89–92), analyzing the lyrics of a meaningful song can assist young people to explore difficult feelings and experiences. Furthermore, Juslin et al. (2008, 2010) purported that imagery and lyrics are two of the psychological mechanisms by which music is related to emotional responses (Juslin and Västfjäll, 2008; Juslin et al., 2010). Thus, the music listening and concurrent activities were designed to enhance participants’ emotional experience during listening in order to increase their skills in recognition of their own emotions and their ability to label and tolerate these emotions.

A pilot study of a 6 h version of *Tuned In* was conducted amongst 50 dysphoric university students aged 18–25 years (67% female). Participants were randomly assigned to *Tuned In* or a wait-list control. *Tuned In* involved groups of around eight participants with two psychologist facilitators. Mixed repeated measures ANOVA results showed that *Tuned In* participants experienced greater improvement in emotional awareness and clarity and total emotion regulation scores than controls. Weekly ratings also indicated significant improvements in emotional awareness, ability to name emotions, and ability to regulate emotions. Ratings of engagement were high and the overall attendance rate was 98% (Dingle and Fay, under review). As such, preliminary data supports the efficacy of this program amongst a non-clinical sample of university students.

The aim of this study was to investigate the effectiveness of *Tuned In* among two adolescent samples in real world educational contexts: a sample of adolescents who had disengaged from school due to learning and psychosocial problems and were enrolled in an experiential learning program run by the non-Government organization BoysTown; and a sample of students at a mainstream girls secondary school in a suburb of a metropolitan city in Australia. It was hypothesized that:

    (1) Participants’ ratings of emotional awareness, ability to name emotions, strategies for regulation and confidence in using their strategies would increase from pre to post intervention;

    (2) Participants would show improvements from pre to post intervention on validated measures of emotion regulation; and

    (3) Participants would find *Tuned In* to be an engaging program, as indicated by their ratings of interest and enjoyment in the program collected at the end of the program.

    (4) That the program could be scaled up from the original small group size of around 8 participants to an en masse school group of over 100 students while remaining effective and engaging.

## Materials and Methods

### Sample 1 – At-Risk Adolescents

#### Participants

Forty-one participants (76% males), aged between 14 and 17 years old (*M*_age_ = 14.83) were recruited from the BoysTown experiential learning program in a regional city in Australia. All consenting members of the program during the study period were recruited to the study. A majority of the sample was born in Australia (88%), with five participants identifying as Aboriginal and Torres Strait Islander and the remaining 12% hailing from New Zealand, the Philippines and Liberia. Participants in this sample experienced multiple barriers to learning and social inclusion resulting in their drop out or exclusion from formal secondary schooling. Furthermore, 18% were reportedly undergoing treatment for a psychological disorder at the time of this study. The sample mean on the K6 (see Measures) was 5.7 (*SD* = 4.9), which is in the normal range, with no participants falling over the cut off of 13 indicative of mental health problems. So although this was not a clinical sample of adolescents, they could be characterized as “at risk” of mental health problems. Less than a quarter of the sample (24.3%) had received a year or more of formal musical training.

### Measures

#### Demographic Information

Participants were asked to report their age in years, sex, ethnicity, and country of origin, and their highest grade completed at school. Musical involvement was assessed with items about music listening engagement, music education and playing.

#### Emotion Variables

At pre- and post-program, participants were asked to rate the extent to which five purpose-written emotion statements were true of them over the past week, on a 7-point Likert type scale from 1 = *Never True*, to 7 = *Always True*. The statements were: *I was aware of what happened in my body when I felt strong emotions*; *I was able to name these feelings (e.g., I’m happy, sad, anxious, etc)*; *I felt confident that I could manage my strong emotions*, *I used music as a way of managing my emotions*, and *I have a range of healthy ways of managing my emotions*. The internal consistency of the emotion variables in sample 1 was α = 0.83 at pre-program and α = 0.90 at post-program.

#### Emotion Regulation Questionnaire

The ERQ (Gross and John, 2003) is a widely used 10-item emotion regulation scale measuring use of cognitive reappraisal (e.g., *When I want to feel more positive emotion (such as joy or amusement), I change what I’m thinking about)*; and suppression (e.g., *I keep my emotions to myself*). Longitudinal research indicates that use of emotional suppression is related to negative social connectedness and wellbeing while cognitive reappraisal is associated with positive social connectedness and wellbeing among young people (English et al., 2012). Participants provided a rating for each item on a 7-point Likert type scale ranging from 1 = *Strongly disagree* to 7 = *Strongly agree*. The internal consistency for the two subscales in the current sample was α = 0.72 and α = 0.61 (for reappraisal and suppression, respectively). As the internal consistency for suppression was low in this sample, the results should be interpreted cautiously.

#### Kessler-6

The K6 is a widely used six-item screen for mental illness designed by Furukawa et al. (2003). The K6 asks respondents to rate how often in the past 30 days they have experienced three symptoms of depression and three symptoms of anxiety. Participants rated their experience of each on a 5-point Likert type scale from 0 = *None of the time* to 4 = *All of the time*, which are summed to a total score in the range of 0 to 24. A cut off score 13 and above is interpreted as indicating a clinical level of distress. The internal consistency of the K6 in sample 1 was α = 0.87.

#### Program Evaluation

Upon completion of the program, three additional items were included to assess responses to the program overall: how helpful participants found the program in helping them to regulate mood; how enjoyable and interesting they found *Tuned In*; and the likelihood they will continue to use music to regulate their emotion. These were rated on a 7 point Likert type scale from 1 = *Never True* to 7 = *Always True*.

### Procedure

Participants took part in the *Tuned In* program as a component of their 2-days a week BoysTown experiential learning program. Participants from five different cohorts were recruited across three terms, with data collection spanning an 8-month period in 2014. Participants completed the online surveys prior to the *Tuned In* program and immediately following the completion of the program. Participants in the BoysTown program did not receive any other psychological intervention during this time, with the curriculum focused solely on literacy, numeracy and life skills. There was a 29% attrition rate during the 10 weeks term, with 29 participants completing the post-program survey (and some measures not being completed accurately). Attrition was due to a range of factors including that the young person started a vocational training program, had to appear in court, or experienced an increase in family difficulties that was associated with their disengagement with the learning program. The *Tuned In* program was facilitated by provisional psychologists enrolled in postgraduate clinical psychology internships at the University of Queensland under the supervision of the first author. Participants received $20 in vouchers for each completed survey as compensation for their time.

### Sample 2 – Mainstream Adolescents

#### Participants

Comprised of 216 female students aged 12 to 15 years (*M*_age_ = 13.6 years) attending an independent school. There were 117 year eight girls and 99 year nine girls. This was a convenience sample in which participants were given a brief survey to complete before and after an en-masse half day *Tuned In* workshop, so only a small selection of variables was assessed. No information was collected on psychological problems or musical training.

#### Emotion Variables

The same emotion variables as collected from Sample 1 were collected at pre- and post-program, with some additional confidence items: *I am confident that I can regulate my anger, I am confident that I can regulate my sadness, I am confident that I can regulate my anxiety, and I am confident that I can be happy without negative consequences.* The internal consistency of these emotion variables in sample 2 was α = 0.75 at pre-program and α = 0.89 at post-program.

#### Program Evaluation

Participants were asked to rate three statements at the end of the program on a 7 point Likert type scale from 1 = *Never True* to 7 = *Always True*: *I enjoyed the Tuned In program*; *I would recommend this program to other students in my year at school*, and *I am likely to continue to use the music emotion regulation strategies learnt in the program*.

### Procedure

The mainstream school study was conducted in two half-day workshops, with all of the year nine girls and then all of the year eight girls together in the school auditorium. The students’ usual teachers were present although they were not directly involved in running the program. Facilitators were the three authors and two other provisionally registered psychologists. This en masse version of the program included key components of the program content such as the two dimensional model of emotion, identifying current and desired emotion on this model, and sharing participants’ music while completing body scan, imagery and lyric analysis activities. On the request of the School leaders, emotions related to three specific themes were emphasized: academic anxiety, relationship problems, and enhancing wellbeing. This was done through the use of age appropriate scenarios asking the students to imagine themselves in the scenario, and how it would make them feel. Data were collected by means of a short pre- and post-program survey completed on paper. Participants were given confectionary as a token of appreciation for their involvement in the study. Methods and procedures were approved by the University Human Research Ethics Committee (approval #2009001748).

## Results

**FIGURE 1:**
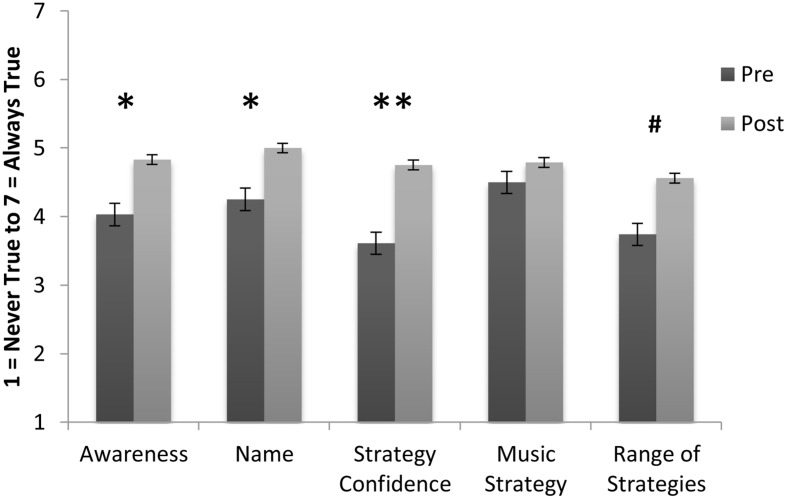
**Sample means from 41 at-risk adolescents on the emotion variables at pre- and post-program.** (Bars are standard errors; significance *p < 0.05, **p < 0.01, ^#^0.10 > *p* > 0.05).

**FIGURE 2:**
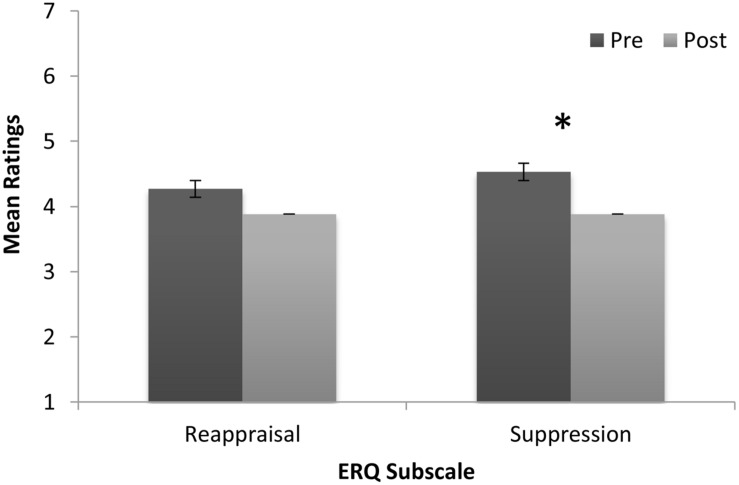
**Sample means for 41 at-risk adolescents on the emotion regulation questionnaire cognitive reappraisal and suppression subscales at pre- and post-program.** (Bars are standard errors; significance *p < 0.05).

Sample 1 means on the emotion variables at pre- and post-program are shown in **Figure 1**. Repeated measures analysis of variance results showed a significant increase in participant’s self-reported emotional awareness from pre to post, *M_pre_* = 4.03, *SD* = 1.59; *M_post_* = 4.83, *SD* = 1.26; *F*(1,28) = 5.28, *p* = 0.029, η^2^ = 0.16. There was also significant increase in their ability to name their emotions: *M_pre_* = 4.25, *SD* = 1.35; *M_post_* = 5.00, *SD* = 1.39; *F*(1,27) = 6.32, *p* = 0.018, η^2^ = 0.19. Furthermore, a significant increase in participants’ confidence to manage their emotions was observed from pre to post, *M_pre_* = 3.61, *SD* = 1.64; *M_post_* = 4.75, *SD* = 1.11; *F*(1,27) = 12.43, *p* = 0.002, η^2^ = 0.32. The use of music to regulate emotions did not change significantly: *M_pre_* = 4.84, *SD* = 1.90, and *M_post_* = 4.90, *SD* = 1.60. Finally, self-reported range of healthy strategies to regulate emotions improved from *M_pre_* = 3.74, *SD* = 1.74 to *M_post_* = 4.56, *SD* = 1.53, however, this change did not quite reach significance: *F*(1,26) = 3.882, *p* = 0.06 (see **Figure 1**). According to scores on the Emotion Regulation Questionnaire, there was a significant decrease in the use of emotional suppression as a regulation strategy from pre- to post-program, *M_pre_* = 4.53, *SD* = 1.02; *M_post_* = 3.88, *SD* = 0.97; *F*(1,24) = 6.44, *p* = 0.018, η^2^ = 0.21. No significant difference was found for the use of reappraisal, *M_pre_* = 4.27, *SD* = 0.86; *M_post_* = 3.77, *SD* = 1.1; *F*(1,24) = 3.35, *p* = 0.08, see **Figure 2**.

Depression and anxiety symptoms on the K6 were in the normal range for the majority of participants and although the sample mean increased slightly from pre- to post-program this did not reach significance, *M_pre_* = 5.64, *SD* = 4.92; *M_post_* = 7.56, *SD* = 4.06; *F*(1,24) = 4.16, *p* = 0.053. Participants rated *Tuned In* to be helpful in managing their emotions (*M* = 5.00, *SD* = 1.6), they found *Tuned In* interesting and enjoyable (*M* = 5.87, *SD* = 1.4) and reported that they were likely to continue to use music to manage their emotions (*M* = 5.45, *SD* = 1.76).

**FIGURE 3:**
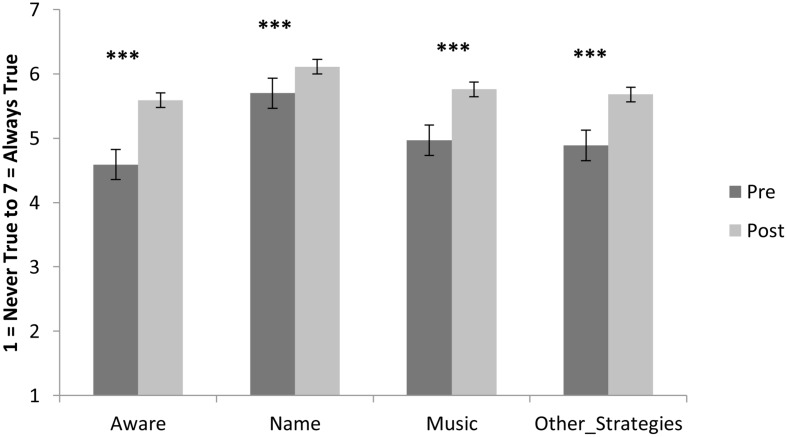
**Sample means for 216 mainstream adolescents on emotion variables at pre- and post-program.** (Bars are standard errors; significance ***p < 0.001).

**FIGURE 4:**
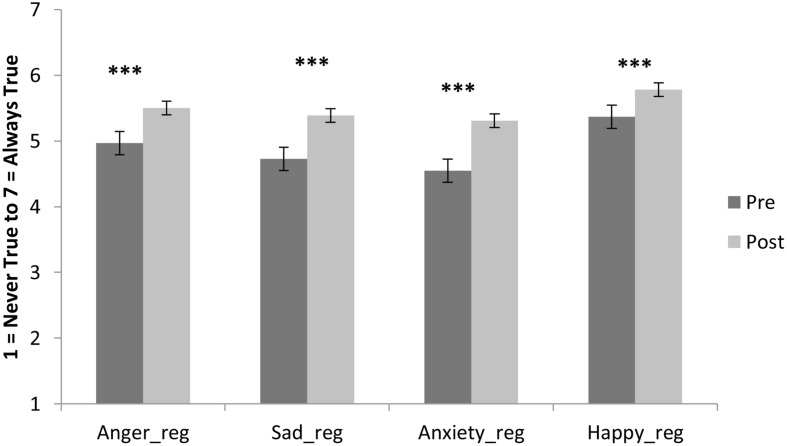
**Sample means for 216 mainstream adolescents on confidence in regulating primary emotions at pre- and post-program.** (Bars are standard errors; significance ***p < 0.001).

The mainstream secondary school sample means on the emotion variables at pre- and post-program are shown in **Figures 3** and **4**. Repeated measures analysis of variance results showed a significant increase in participants’ emotional awareness from *M_pre_* = 4.59, *SD* = 1.44 to *M_post_* = 5.59, *SD* = 1.23; *F*(1,213) = 103.13, *p* < 0.001, η^2^ = 0.326. There was also a significant increase participants’ affective labeling of their emotions, *M_pre_* = 5.70, *SD* = 1.10; *M_post_* = 6.11, *SD* = 1.02; *F*(1,214) = 27.33, *p* < 0.001, η^2^ = 0.113. The use of music to regulate emotions increased from *M_pre_* = 4.97, *SD* = 1.83 to *M_post_* = 5.76, *SD* = 1.43; *F*(1,212) = 43.655, *p* < 0.001, η^2^ = 0.171. Furthermore, participants’ range of healthy strategies to regulate emotions improved from *M_pre_* = 4.89, *SD* = 1.46 to *M_post_* = 5.68, *SD* = 1.31, *F*(1,213) = 63.52, *p* < 0.001, η^2^ = 0.230. Confidence in regulating anger increased from *M_pre_* = 4.97, *SD* = 1.55 to *M_post_* = 5.50, *SD* = 1.42, *F*(1,210) = 30.76, *p* < 0.001, η^2^ = 0.128. Confidence in regulating sadness improved from *M_pre_* = 4.73, *SD* = 1.52 to *M_post_* = 5.39, *SD* = 1.46, *F*(1,215) = 55.187, *p* < 0.001, η^2^ = 0.204. Anxiety regulation confidence increased from *M_pre_* = 4.55, *SD* = 1.79 to *M_post_* = 5.31, *SD* = 1.49, *F*(1,211) = 52.36, *p* < 0.001, η^2^ = 0.199. Finally, confidence that they could be happy without negative consequences was rated *M_pre_* = 5.37, *SD* = 1.52, and *M_post_* = 5.78, *SD* = 1.21, *F*(1,212) = 22.067, *p* < 0.001, η^2^ = 0.094. The program was rated an average of 5.85 (*SD* = 1.26) out of 7 for enjoyment, 5.61 (*SD* = 1.42) for recommended to other students, and 5.86 (*SD* = 1.27) for likelihood of continuing to use the emotion regulation strategies learned.

## Discussion

The aim of this study was to assess the effectiveness of the group program *Tuned In* to enhance emotion awareness and regulation skills of at-risk and mainstream adolescents in educational settings. The at-risk adolescents were offered an eight session version of the program in groups of around eight participants and showed significant improvements in self-reported emotional awareness, affective labeling of their own emotions, and confidence in using a range of emotion regulation strategies. Interestingly, the use of music as a strategy for regulating emotions did not increase from pre- to post-program, however, scores were highest at pre-program for this variable. This finding is consistent with previous research showing that adolescents are high consumers of music and use music listening naturally as a way of managing their emotions (Saarikallio and Erkkilä, 2007; Australian Psychological Society, 2015; Papinczak et al., 2015).

Further supporting these improvements in emotion variables, scores on the validated emotion regulation questionnaire showed that participants used the maladaptive strategy of emotional suppression significantly less at post-intervention. No significant change was observed in the use of adaptive, cognitive reappraisal. This pattern of results made sense given that the *Tuned In* program did not provide participants with cognitive-based strategies for emotion regulation, instead encouraging participants to explore emotional experiences using music. With participants encouraged to notice and then share the physical sensations and imagery that arose during the course of a song, they were being exposed to their emotions in a safe environment (Baker et al., 2007). It follows that scores on emotional suppression, a strategy aimed at preventing emotional expression, decreased at post-intervention as the expression of participant’s emotional experiences had been normalized and validated through the *Tuned In* program. Research suggests that ongoing emotional suppression plays a central role in the development of social problems (English et al., 2012) and psychopathology (Aldao and Nolen-Hoeksema, 2012), with the use of maladaptive strategies proposed to be more harmful than the absence of adaptive strategies. These results are encouraging as they lend support to the effectiveness of the program as a preventative intervention.

The improvement in measures of emotion awareness and regulation did not translate to symptom change on the K6, although there was limited room for improvement as the mean was in the normal range at pre-program. In fact, there was a slight increase in the K6 mean from pre- to post-program although this could be accounted for by the usual fluctuations over time within the non-clinical range on this measure. It failed to reach statistical significance or move into the clinical range (of 13 or above using the suggested cut off score given in Kessler et al., 2003). Further research with adolescents who experience clinical levels of depression and anxiety is necessary to test whether the emotion regulation training translates to mood symptom improvement. Further research is also necessary to establish whether the emotional skills learned in the program generalized to the home or other environments and were sustained over time – questions which were unfortunately beyond the scope of the current study. Importantly, given that these adolescents were disengaged from mainstream school and many experienced social disadvantage and exclusion, they rated the *Tuned In* program as interesting and enjoyable, and that they were likely to continue to use the strategies taught in the program. This indication that a music based program was engaging to young people has been found in other research, for instance, in educational contexts (Cheong-Clinch, 2009) and in clinical contexts (Dingle et al., 2008). Taken together, this pattern of results for the emotion variables provides proof of concept that the *Tuned In* program enhances at-risk adolescents’ emotional skills.

In the mainstream school sample, despite the limited opportunity for individual participants to share their music and to discuss their emotional experiences in this en masse format, results indicated that *Tuned In* was effective at improving adolescents’ confidence in regulating a range of emotions, and enhanced their self-reported emotional awareness and regulation strategies. Although the emotion variable means were higher at pre-program in the mainstream sample (around 4.5 to 5.5 out of 7, see **Figures 3** and **4**) than in the at-risk sample (around 4, see **Figure 1**), a significant improvement was also seen across all emotion measures in the mainstream sample. Of all of the primary emotions assessed, students reported the lowest confidence in regulating their anxiety at pre-program, and this was one theme that the program particularly attended to. Further research is required to ascertain whether this increase in anxiety regulation confidence at post-program might translate to decreases in academic anxiety around assignment and exam time, and improvements in performance across academic, sporting, and music domains where anxiety can be a barrier to performance (Osborne, 2012; Ringeisen and Raufelder, 2015).

The program ratings indicated that participants in the mainstream school found it to be an interesting and engaging program that they would recommend to same age peers. Furthermore, they rated their likelihood to continue to use the strategies learned in the program as high, although because the workshops were conducted in the final week of the school year, we were unable to conduct a follow up assessment to confirm this. The effectiveness on emotion variables and the program ratings lend support for the fourth hypothesis that the program can be scaled up to large school groups, which makes it a cost effective intervention.

Overall, the findings of this study indicate support for the four hypotheses and provide a proof of concept that the *Tuned In* program enhances emotion awareness and regulation of adolescents in educational settings. However, there were a number of limitations to the study. Due to practical and resource limitations there were no control groups so our conclusions are based on within group changes only. The lack of follow up means we can’t account for whether any gains were maintained over time. The Emotion Regulation Questionnaire subscale scores used in sample 1 had low to moderate internal consistency reliability values. Further analysis indicated that participants did not respond especially differently to any one item on the subscales (that is, item deletion did not significantly improve the Cronbach’s alpha values). However, the participants may have found the scale difficult to understand due to the meta-cognitive nature of the items, e.g., “when I want to feel more positive emotion, I change what I am thinking about”. It would therefore be recommended that future research with at-risk adolescents use a different measure of emotion regulation that is less reliant on meta-cognitive awareness. Alongside of the measure of emotion regulation, future research would need to include broader measures of social and academic functioning – potentially including teacher and parent reports to address the issue of demand characteristics on participants’ self-reports.

Questions that remain to be investigated include: what are the moderators and mediators of the effectiveness of *Tuned In*? Other research shows that individual factors such as reward sensitivity, emotional sensitivity to music and absorption in music contribute to its effect on listeners (Sandstrom and Russo, 2011; Loxton et al., 2016). Several recent studies have found that a tendency to ruminate or brood influences whether people listen to music in ways that are helpful or unhelpful to their mood (McFerran and Saarikallio, 2013; Garrido and Schubert, 2015; McFerran, 2016). Music with lyrics may have a particularly powerful influence on the mood of adolescents who score more highly in ruminative thoughts than adults of older age groups (Sütterlin et al., 2012). In respect to sad music in particular, variations in the ways people conceptualize sadness and music lead to differences in the emotion regulation processes at play (Peltola and Eerola, 2016). It would be interesting to investigate the mechanisms through which music influenced participants’ emotions in *Tuned In* beyond those specifically targeted in the program (appraisal of lyrics, imagery, and bodily sensations). Other mechanisms have been described in work by Juslin et al. (2008, 2010) such as emotional contagion and evaluative conditioning to the music.

## Conclusion

The findings of this study show evidence that *Tuned In* helps build emotional awareness and regulation amongst at-risk and mainstream adolescents, and results are consistent with previous findings in a young adult sample (Dingle and Fay, under review). Adolescence is a time of growth, change, and emotional upheaval during which individuals’ capacity for self-regulation of emotion is still developing. The *Tuned In* program is engaging and effective at enhancing participants’ emotional skills in small and large groups.

## Author Contributions

GD developed the intervention program, designed the study, set up the collaboration with the stakeholders of Sample 1, and took overall responsibility for the manuscript. JH set up the collaboration with the stakeholders of Sample 2 and was involved in facilitating the program with both Samples, data collection, and input into the written manuscript. AK was involved with facilitating the program with both Samples, data collection and analysis of Sample 1 data, and input into the written manuscript.

## Conflict of Interest Statement

The authors declare that the research was conducted in the absence of any commercial or financial relationships that could be construed as a potential conflict of interest.
